# Proteomic analysis of serum extracellular vesicles reveals Fibulin-3 as a new marker predicting liver-related events in MASLD

**DOI:** 10.1097/HC9.0000000000000448

**Published:** 2024-06-03

**Authors:** Sadatsugu Sakane, Hayato Hikita, Kumiko Shirai, Tatsuya Sakamoto, Ryohei Narumi, Jun Adachi, Naruyasu Kakita, Yukinori Yamada, Hidenori Toyoda, Hirokazu Takahashi, Goki Suda, Machiko Kai, Yuki Tahata, Ryotaro Sakamori, Shusuke Kumazaki, Kenji Fukumoto, Yuta Myojin, Kazuhiro Murai, Takahiro Kodama, Tomohide Tatsumi, Takeshi Tomonaga, Naoya Sakamoto, Eiichi Morii, Tetsuo Takehara

**Affiliations:** 1 Department of Gastroenterology and Hepatology, Graduate School of Medicine, Osaka University, Osaka, Japan; 2 Laboratory of Proteomics for Drug Discovery, Center for Drug Design Research, National Institute of Biomedical Innovation, Health and Nutrition, Osaka, Japan; 3 Department of Gastroenterology and Hepatology, Kaizuka City Hospital, Osaka, Japan; 4 Department of Gastroenterology, Ogaki Municipal Hospital, Ogaki, Japan; 5 Liver Center, Saga University Hospital, Saga, Japan; 6 Department of Gastroenterology and Hepatology, Graduate School of Medicine, Hokkaido University, Sapporo, Japan; 7 Department of Pathology, Osaka University Graduate School of Medicine, Osaka, Japan

## Abstract

**Background:**

There is a need for novel noninvasive markers for metabolic dysfunction–associated steatotic liver disease (MASLD) to stratify patients at high risk for liver-related events including liver cancer and decompensation. In the present study, we used proteomic analysis of proteins in extracellular vesicles (EVs) to identify new biomarkers that change with fibrosis progression and can predict the development of liver-related events.

**Methods:**

We analyzed serum EVs from 50 patients with MASLD assessed for liver fibrosis by biopsy and identified proteins that altered with advanced fibrosis. A further evaluation was conducted on another cohort of 463 patients with MASLD with biopsy.

**Results:**

Eight candidate proteins were identified by proteomic analysis of serum EVs. Among them, serum levels of Fibulin-3, Fibulin-1, and Ficolin 1 correlated with their EV levels. In addition, serum Fibulin-3 and serum Fibulin-1 levels changed significantly with advanced fibrosis. Using another cohort with biopsy, we found that the serum Fibulin-3 concentration was significantly greater in those with advanced fibrosis but that the serum Fibulin-1 concentration was not significantly different. Multivariate Cox proportional hazards analysis revealed that a higher Fibrosis-4 (FIB-4) index and higher serum Fibulin-3 concentration were independent risk factors for liver-related events. When the cutoff value for the serum Fibulin-3 concentration was 6.0 µg/mL according to the Youden index of AUROCs, patients with high serum Fibulin-3 significantly more frequently developed liver-related events than did other patients. Validation using another cohort of 226 patients with clinically diagnosed MASLD confirmed that high serum Fibulin-3 levels are associated with a greater frequency of liver-related events.

**Conclusions:**

Serum Fibulin-3 was identified as a biomarker for predicting liver-related events in patients with MASLD.

## INTRODUCTION

NAFLD is the most common chronic liver disease, with an incidence of 38% worldwide and 30% in East Asia.^1^ Some patients with NAFLD develop liver-related events, such as liver cancer and decompensation, which require hospitalization and treatment.^1^ As the frequency of liver-related events in patients with NAFLD is relatively low compared to that in patients with viral hepatitis,^2^ screening for liver-related events, such as abdominal echocardiography and gastrointestinal endoscopy, at a high frequency in all patients with NAFLD is not desirable in terms of compliance or cost-effectiveness.

In general, liver fibrosis is an independent and strong prognostic factor for liver-related events, including liver cancer and decompensated liver events, in patients with NAFLD.^3^,^4^,^5^,^6^,^7^ The gold standard for evaluating liver fibrosis is liver biopsy. However, it is not advisable to perform liver biopsy for all patients with NAFLD, and liver fibrosis can be evaluated using noninvasive methods, including serum markers or a scoring system using serum markers.^8^ Indeed, several serum fibrosis markers and scoring systems have been reported to predict the development of liver-related events.^9^,^10^,^11^,^12^ In addition to the use of serum fibrosis markers or scoring systems, liver stiffness has been reported to be useful in predicting liver-related events.^13^ Nevertheless, the ability of these noninvasive markers to predict liver-related events is insufficient. Accordingly, there is a significant demand for the discovery and development of new markers.

Proteomics is a useful tool in the search for novel biomarkers and is widely used, but its practical application is very rare.^14^ Although several studies have explored biomarkers for NAFLD using serum proteomic analysis,^15^ there are no markers that can sensitively predict liver-related events. In a different approach than has been used in other studies, we focused on extracellular vesicles (EVs). EVs are cell-derived vesicles surrounded by a lipid bilayer ranging from 30 to 2000 nm in diameter^16^ that are involved in many physiological and pathological processes.^17^ There are many proteins in serum EVs, and proteins present in minute amounts in serum may accumulate in EVs. It has been reported that more proteins are identified in serum EVs than in serum when proteomics is performed.^18^ Enrichment of low-abundance proteins, which usually remain unidentified through conventional serum proteomic analysis, allows for searching for additional proteins in a comprehensive manner.^19^ On the other hand, extraction of serum EVs is time-consuming and expensive, making it unsuitable for widespread clinical use. Therefore, we thought it would be useful to search for new serum markers by using serum EVs to narrow down the candidate proteins for markers and to pick up candidate proteins whose content in serum EVs correlates with the amount of protein in serum. Indeed we identified novel serum markers of liver fibrosis in patients with chronic hepatitis C by this method.^20^ In the present study, we applied proteomics of serum EVs to identify novel serum biomarkers that reflect liver fibrosis.

Recently, metabolic dysfunction–associated steatotic liver disease (MASLD) was proposed as a new disease concept; this disease is defined as steatotic liver that meets cardiometabolic criteria with no other factors that cause steatotic liver.^21^,^22^,^23^ In the present study, we searched for novel fibrosis markers that can accurately predict the development of liver-related events in patients with MASLD. We first selected proteins that reflect liver fibrosis as candidate novel markers for predicting liver-related events in patients with MASLD and then examined whether the candidate proteins identified are useful for predicting the development of liver-related events.

## METHODS

### Patient characteristics

Three cohorts were used in this study. In total, 50 consecutive patients diagnosed with NAFLD by liver biopsy at Osaka University Hospital between 2016 and 2020 were enrolled in cohort 1 (Supplemental Figure S1A, http://links.lww.com/HC9/A893). A total of 476 patients diagnosed with NAFLD by liver biopsy (180 patients from Ogaki Municipal Hospital between 2005 and 2021, 170 patients from Kaizuka City Hospital between 2014 and 2020, and 126 patients from Saga University Hospital between 2012 and 2021) were enrolled in cohort 2 (Supplemental Figure S1B, http://links.lww.com/HC9/A893). A total of 228 patients who were clinically diagnosed with NAFLD at Hokkaido University Hospital between 2002 and 2020 were enrolled in cohort 3 (Supplemental Figure S1C, http://links.lww.com/HC9/A893).

We defined a liver-related event as the occurrence of either liver cancer or decompensation. A liver cancer event was defined as the first occurrence of liver cancer after liver biopsy. A decompensating event was defined as the development of esophageal varices, gastric varices, ascites, or HE.

As the aim of this study was to identify biomarkers for liver-related events in patients with MASLD, patients with other chronic liver diseases and those with a history of developing liver-related events were excluded. To select only those patients who fulfill MASLD diagnostic criteria, we also excluded patients lacking evidence of any cardiometabolic risk factor, in accordance with the Delphi consensus.^21^,^22^,^23^ As a result, a total of 50 patients were included in cohort 1, 473 patients were included in cohort 2, and 226 patients were included in cohort 3 (Supplemental Figure S1A, http://links.lww.com/HC9/A893, Supplemental Figure S1B, http://links.lww.com/HC9/A893, Supplemental Figure S1C, http://links.lww.com/HC9/A893).

This study was approved by all 5 participating institutes. All patients provided informed consent, and the study design adhered to the tenets of the Declaration of Helsinki. The protocol for the study using patient serum was approved by the Institutional Review Board Committees of Osaka University Hospital (Institutional Review Board No. 17032).

### Clinical samples and clinical information

EVs were extracted from the serum of the 50 patients in cohort 1 (Supplemental Table S1, http://links.lww.com/HC9/A893) at the time of liver biopsy, and proteomic analysis was performed. The same sera were also used to measure serum protein levels through ELISA. Serum Fibulin-3 and Fibulin-1 levels of the 473 patients in cohort 2 (Table 2) were measured at the time of liver biopsy and compared with the clinical course. Serum Fibulin-3 levels of the 226 patients in cohort 3 (Supplemental Table S2, http://links.lww.com/HC9/A893) were measured at the time of diagnosis, and the clinical course was evaluated in the same manner as for cohort 2.

### Histological assessment

Histologic diagnosis was made by liver biopsy for all patients. Liver samples were embedded in paraffin blocks and stained with hematoxylin and eosin and Azan. All liver biopsies for this study were evaluated centrally by experienced pathologists belonging to the Department of Pathology, Osaka University Graduate School of Medicine. Hepatic fibrosis was graded as F0-F4 according to the Kleiner classification^24^; F3 and F4 were regarded as advanced fibrosis. Hepatic steatosis, lobular inflammation, and hepatocellular ballooning were also scored according to the Kleiner classification.^24^ The nonalcoholic fatty liver disease activity score was calculated as the sum of the steatosis, lobular inflammation, and ballooning scores.

### Isolation and solubilization of EVs from patient sera

Isolation and solubilization of EVs from patient sera were performed through the same method as we reported.^20^ EVs were isolated by an affinity-based method using Magcapture Exosome Isolation Kit PS (catalog number; 293-77601, FUJIFILM Wako Pure Chemical Corporation).^25^ We have reported the validity of this method of extracting EVs from serum in a another publication^26^ with reference to the guidelines.^27^

### Proteomic analysis

Each sample was solubilized with phase-transfer surfactant buffer^28^ followed by reduction, alkylation, tryptic digestion, and desalting using StageTips.^29^ LC-MS/MS analysis of EVs was performed using the data-independent acquisition mass spectrometry (DIA-MS) method^30^ based on a published method,^26^ with minor modifications. Peptides were separated on an analytical column (75 μm×20 cm, packed in-house with ReproSil-Pur C18-AQ, 1.9 μm resin; Dr Maisch), and separation was achieved using a 45-minute gradient of 5%–30% acetonitrile in 0.1% formic acid at a flow rate of 280 nL/min. Data were acquired using the data-independent acquisition mode (DIA). An Orbitrap Fusion Lumos mass spectrometer (Thermo Fisher Scientific) was used for gas-phase fractionation-DIA acquisition of a pooled sample for the library, and full mass spectra were acquired with the following parameters: a 2 *m/z* isolation window, a resolution of 120,000, an automatic gain control (AGC) target of 1×10^6^ ions with a 250 ms maximum injection time, and a normalized collision energy of 30. The 5 gas-phase fractionation-DIA runs collectively covered 418–782 *m/z* (ie, 418–494, 490–566, 562–638, 634–710, and 706–782 *m/z*). MS2 spectra were collected with the following parameters: a 2-*m/z* isolation window at 50,000 resolution, an AGC target of 2×10^5^ ions, a maximum injection time of 86 ms, and a normalized collision energy of 30. For the individual samples used for proteome profiling, full mass spectra were acquired in the range of 420–780 *m/z* with the following parameters: a resolution of 120,000 and an AGC target of 4×10^5^ with a maximum injection time of 100 ms. MS2 spectra were collected with the following parameters: a 10-*m/z* isolation window at 30,000 resolution, an AGC target of 2×10^5^ ions, a maximum injection time of 54 ms, and a normalized collision energy of 30. The MS data (raw file) were processed with Spectonaut (ver. 13.9). The database search included all entries from the *Homo sapiens* UniProt database (downloaded in January 2018; taxonomy ID: 9606) and the contaminant database. The search parameters were as follows: up to 2 missed cleavage sites, 7–52 peptide length, carbamidomethylation of cysteine as a static modification, acetyl (protein n-term), and oxidation of methionine as variable modifications. Global normalization using the median value was performed in this process.

### Enzyme-linked immunosorbent assay

The amount of each protein in serum was measured using commercially available ELISA kits, as follows: Circulex Human Fibulin-3/EFEMP1 ELISA Kit (catalog number: CY-8120; MBL); Human MASP1 (Mannan Associated Serine Protease 1) ELISA Kit (catalog number: MBS2507077/96; MyBioSource, Inc.); CircuLex Human Fibulin-1 ELISA Kit Ver. 2 (catalog number: CY-8094V2; MBL); Human Gelsolin ELISA Kit (catalog number: ab270215; Abcam); Human Dermcidin (DCD) ELISA Kit (catalog number: MBS2704747/96; MyBioSource, Inc.); Human Na (+)/H (+) exchange regulatory cofactor NHE-RF2 ELISA Kit (catalog number: MBS9326257/96; MyBioSource, Inc.); Human Mast cell-expressed membrane protein 1, C19orf59 ELISA Kit (catalog number: MBS1606539/96; MyBioSource, Inc.); and Human FCN1/M-Ficolin ELISA Kit (catalog number: ab213777; Abcam).

### Measurement of mRNA expression in liver tissue

Liver tissue was used from 32 of the 50 cases in cohort 1 for which frozen tissue was available. Total RNA was extracted from the samples using an RNeasy Micro Kit (catalog number: 74004; Qiagen). Four hundred nanograms of total RNA eluted from each sample was used for reverse transcription using a ReverTra Ace qPCR RT Kit (catalog number: FSQ-101; Toyobo). The mRNA expression of specific genes was analyzed using a TaqMan Gene Expression Assay (Thermo Fisher Scientific). TaqMan PCR probes were obtained from Thermo Fisher Scientific (human actin be-ta; Hs01060665_g1, human EFEMP1; Hs00244575_m1).

### Data analysis and statistics

Data are presented as the mean ± SD. Comparisons between the 2 groups were performed with the Mann-Whitney *U* test. Categorical data were analyzed by the Fisher exact test. Correlations were assessed by the Pearson product-moment correlation coefficient. The difference in cumulative event rates between the 2 groups was assessed by the log-rank test. Independent factors associated with event occurrence were examined using Cox proportional hazards models. The diagnostic performance for predicting clinical outcomes was assessed by the AUROC curve. AUROCs were compared by using the Delong test. Statistical significance was indicated by *p* < 0.05. Most analyses were performed with R (version 4.1.2)^31^ and R Studio (version 2022.07.2)^32^ software. The Rcmdr (version 2.7-1) package and the BiocManager (version 1.30.16) package were used for the Mann-Whitney *U* test, Fisher exact test, log-rank test, and Cox proportional hazard models. A heatmap was generated using the ComplexHeatmap package (version 2.8.0) in R Studio. DeLong tests were performed by using the pROC (version 1.17.0.1) package. Other statistical analyses and graphs were created using Prism (version 9.4.1) for Windows.

## RESULTS

### Comprehensive search for serum markers that change with fibrosis progression in patients with MASLD through proteomic analysis of serum EVs

**FIGURE 1:**
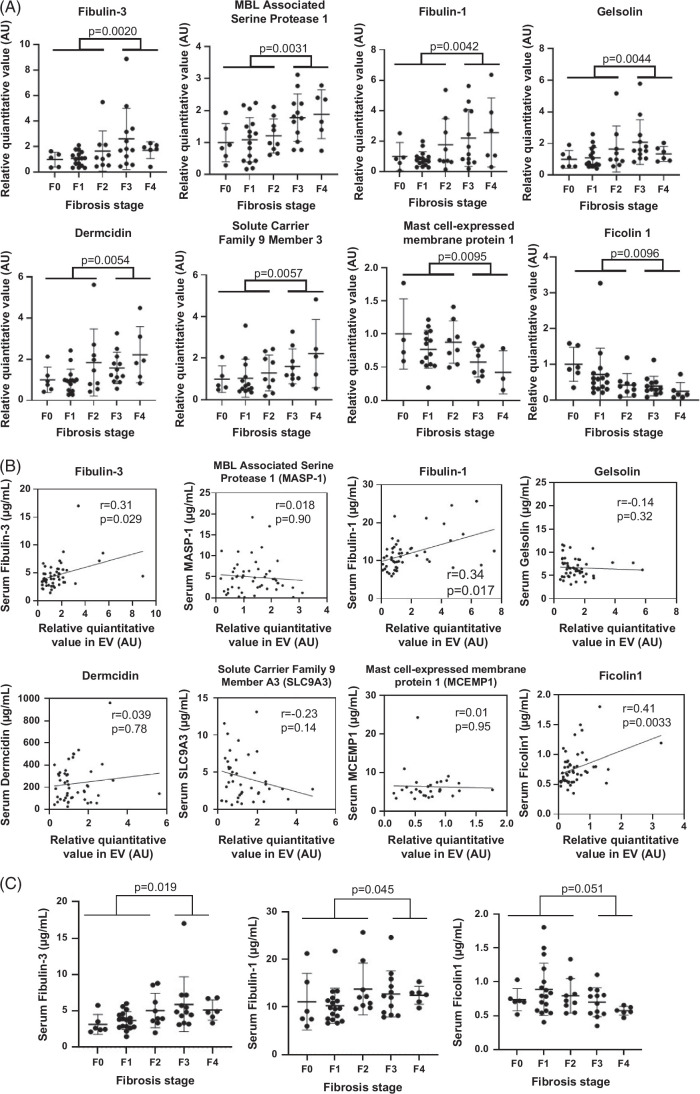
Proteins that change with advanced fibrosis according to MASLD criteria were identified through proteomic analysis of serum extracellular vesicles. (A) Relative levels of 8 proteins in serum EVs according to Brunt stage in 50 patients with MASLD; levels were significantly different between F3-F4 and F0-F2. (B) Correlations between the candidate proteins in serum EVs and those in serum. (C) Relative serum Fibulin-3, serum Fibulin-1, and serum Ficolin 1 levels in patients according to fibrosis stage. The dot plot shows individual values. Data are expressed as the mean ± SD. The difference between the 2 groups was assessed by the Mann-Whitney *U* test. Correlations were assessed by the Pearson product-moment correlation coefficient. Abbreviations: EV, extracellular vesicle; MASLD, metabolic dysfunction–associated steatotic liver disease.

First, proteomic analysis of serum EVs was performed using cohort 1 (50 patients with MASLD assessed for liver fibrosis by biopsy) to identify candidate biomarkers associated with the pathogenesis of MASLD. In this cohort, patients with advanced fibrosis (F3-F4) were older, had lower platelet and serum cholinesterase levels, and had higher total cholesterol and various fibrosis markers than patients with nonadvanced fibrosis (Supplemental Table S1, http://links.lww.com/HC9/A893). DIA-MS identified 1459 proteins in the serum EVs of these 50 patients (Supplemental Figure S2A, http://links.lww.com/HC9/A893). We compared the difference in protein expression between patients with advanced and nonadvanced fibrosis (Supplemental Figure S2B, http://links.lww.com/HC9/A893), and 8 proteins were significantly different between the 2 groups (Mann-Whitney *U* test *p* < 0.01) (Figure 1A, Table 1).

**TABLE 1:** Biomarker candidate proteins that change with fibrosis progression in patients with MASLD

	F3-F4 vs. F0-F2	
Protein name	Log2 fold change	Univariate *p*
Fibulin-3	0.796	0.0020
MBL Associated Serine Protease 1	0.711	0.0031
Fibulin-1	1.049	0.0042
Gelsolin	0.634	0.0044
Dermcidin	0.868	0.0054
Solute Carrier Family 9 Member A3	1.049	0.0057
Mast cell-expressed membrane protein 1	−0.878	0.0095
Ficolin 1	−0.784	0.0096

*Note*: Comparisons between the 2 groups were performed with the Mann-Whitney *U* test.

Abbreviation: MASLD, metabolic dysfunction–associated steatotic liver disease.

Since serum itself used as a biomarker is a simpler and lower cost than serum EV, the correlation between these protein levels in serum EVs and their serum concentration was examined (Figure 1B). Three of the 8 proteins, Fibulin-3, Fibulin-1, and Ficolin 1, exhibited significant correlations between EVs and serum levels (Pearson product-moment correlation coefficient *p* < 0.05). Among these 3 proteins, Fibuiln-3 and Fibulin-1 were significantly different between patients with advanced and nonadvanced fibrosis (Mann-Whitney *U* test *p*<0.05) (Figure 1C).

### The serum Fibulin-3 concentration correlates with several fibrosis markers in patients with MASLD

**FIGURE 2:**
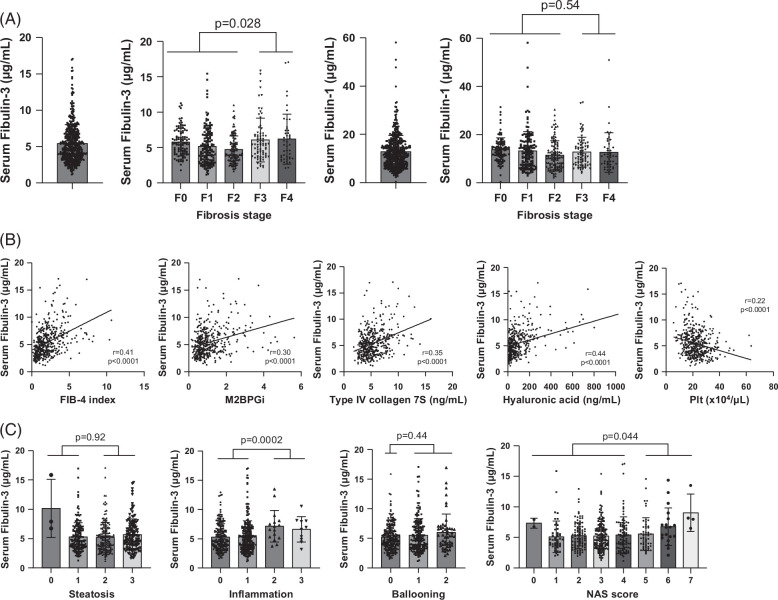
The serum Fibulin-3 concentration increased in patients with advanced fibrosis according to the MASLD criteria. (A) Relative quantitative values of serum Fibulin-3 and serum Fibulin-1 according to fibrosis stage in 476 patients with MASLD. (B) Correlations between the serum Fibulin-3 concentration and fibrosis markers. (C) Relative quantitative values of serum Fibulin-3 according to steatosis, inflammation, ballooning, and NAS score in 476 patients with MASLD. The dot plot shows individual values. Data are expressed as the mean ± SD. The difference between the 2 groups was assessed by the Mann-Whitney *U* test. Correlations were assessed by the Pearson product-moment correlation coefficient. Abbreviations: FIB-4, Fibrosis-4; MASLD, metabolic dysfunction–associated steatotic liver disease; NAS, nonalcoholic fatty liver disease activity score.

To examine whether serum Fibulin-3 and Fibulin-1 levels change with advanced fibrosis in another cohort, we measured the serum Fibulin-3 and serum Fibulin-1 levels in cohort 2 (473 patients with MASLD with biopsy). The median serum Fibulin-3 concentration was determined to be 4.9 µg/mL, ranging from 1.2 to 17.0 µg/mL; the median serum Fibulin-1 concentration [range] was 11.8 µg/mL [0.3–58.1 µg/mL] (Figure 2A). The serum Fibulin-3 concentration was greater in patients with advanced fibrosis than in patients with nonadvanced fibrosis, with no change in the serum Fibulin-1 concentration between patients with advanced and nonadvanced fibrosis (Figure 2A). When patients in cohort 2 were divided using a median cutoff of 4.9 µg/mL, those in the high serum Fibulin-3 subgroup were older, more often female, had lower platelet counts and serum albumin levels, had higher serum type IV collagen 7S, hyaluronic acid, Mac-2-binding protein glycan isomer (M2BPGi) levels, and had a higher Fibrosis-4 (FIB-4) index (Table 2). The serum Fibulin-3 concentration correlated positively with the FIB-4 index, M2BPGi concentration, type IV collagen 7S concentration, and hyaluronic acid concentration and negatively with platelet count (Figure 2B). The ROC curve predicting fibrosis stage 3 or higher was examined (Supplemental Figure S2C, http://links.lww.com/HC9/A893). The AUROC for Fibulin-3 was 0.568, which was similar to 0.588 for M2BPGi (Delong test *p*=0.29), and lower than 0.652 for the FIB-4 index, 0.656 for type IV collagen 7S, 0.632 for hyaluronic acid, and 0.642 for platelet counts (Delong test *p*<0.05). Next, we examined whether serum Fibulin-3 was associated with pathological findings including steatosis, inflammation, and ballooning. Although there is no association with Fibulin-3 for steatosis or ballooning, cases of inflammation 2 or 3 had higher levels of Fibulin-3 than cases of inflammation 0-1. Cases of nonalcoholic fatty liver disease activity score 5–7 had higher levels of Fibulin-3 than cases of nonalcoholic fatty liver disease activity score 0–4 (Figure 2C). Since serum Fibulin-3 was suggested to be associated with liver fibrosis and intrahepatic inflammation, we examined Fibulin-3 mRNA expression in the liver using liver tissue from 32 of the 50 cases in cohort 1 for which frozen tissue was available. There was a significant correlation between serum Fibulin-3 and Fibulin-3 mRNA/β actin mRNA ratio in the liver (Supplemental Figure S2D, http://links.lww.com/HC9/A893).

**TABLE 2:** Characteristics of 476 patients with MASLD (cohort 2)

	All (N=473)	Fibulin-3 low (N=237)	Fibulin-3 high (N=236)	*p*
Age (y)	58 [11–87]	51 [11–84]	65 [17–87]	<0.0001
Sex (male/female)	200/273	124/113	76/160	<0.0001
BMI (kg/cm^2^)	27.9 [16.8–53.3]	28.2 [18.2–53.3]	27.6 [16.8–48.9]	0.21
Diabetes mellitus (yes/no)	244/229	134/103	110/126	0.031
Plt (×10^3^/µL)	225 [56–637]	239 [94–506]	212 [56–637]	0.00011
Alb (g/dL)	4.3 [1.9–5.2]	4.4 [1.9–5.2]	4.3 [2.9–5.1]	0.0016
T-Bil (mg/dL)	0.8 [0.2–2.9]	0.8 [0.2–2.3]	0.8 [0.2–2.9]	0.79
AST (U/L)	59 [15–300]	56 [15–300]	62 [19–245]	0.050
ALT (U/L)	81 [12–501]	88 [14–501]	75 [12–275]	0.015
γGTP (U/L)	87 [12–1070]	87 [12–1070]	86 [14–431]	0.88
AFP (ng/mL)	4.4 [0.7–93]	3.9 [0.8–93]	4.9 [0.7–41]	0.056
HbA1c (%)	6.3 [4.2–15.7]	6.1 [4.2–11.6]	6.4 [4.2–15.7]	0.024
TG (mg/dL)	167 [38–605]	174 [38–605]	160 [49–530]	0.098
LDL-Chol (mg/dL)	123 [34–254]	125 [34–254]	121 [50–229]	0.21
Type 4 Collagen 7S (ng/mL)	5.4 [1.8–16.0]	4.8 [1.8–11.1]	6.0 [2.6–16.0]	<0.0001
Hyaluronic acid (ng/mL)	106 [7–3099]	57 [7–538]	150 [9–3099]	<0.0001
M2BPGi (COI)	1.1 [0.2–5.6]	0.9 [0.2–4.2]	1.3 [0.2–5.6]	<0.0001
FIB-4 index	2.09 [0.17–10.62]	1.50 [0.17–6.33]	2.68 [0.31–10.62]	<0.0001
Fibrosis stage (0/1/2/3/4)	103/145/106/73/46	40/79/66/31/21	63/66/40/42/25	0.0054
Liver cancer occurrence (yes/no)	17/456	3/234	14/222	0.0064
Decompensation event (yes/no)	18/455	3/234	15/221	0.0038
Liver-related event (yes/no)	25/448	4/233	21/215	0.00046
Observation period (y)	6.2 [0.04–17.4]	6.2 [0.08–17.4]	6.2 [0.04–16.8]	0.97
Fibulin-3 (μg/mL)	5.4 [1.2–17.1]	3.5 [1.2–4.9]	7.3 [4.9–17.0]	<0.0001

*Note*: Comparisons between the 2 groups were performed with the Mann-Whitney *U* test. Categorical data were analyzed by the Fisher exact test.

Abbreviations: γGTP, Gammna-glutamyl transpeptidase; AFP, alpha-fetoprotein; FIB-4, Fibrosis-4; LDL-Chol, low-density lipoprotein cholesterol; MASLD, metabolic dysfunction–associated steatotic liver disease; TG, triglyceride.

### The serum Fibulin-3 concentration and FIB-4 index are independent predictors of liver-related events in patients with MASLD

**FIGURE 3:**
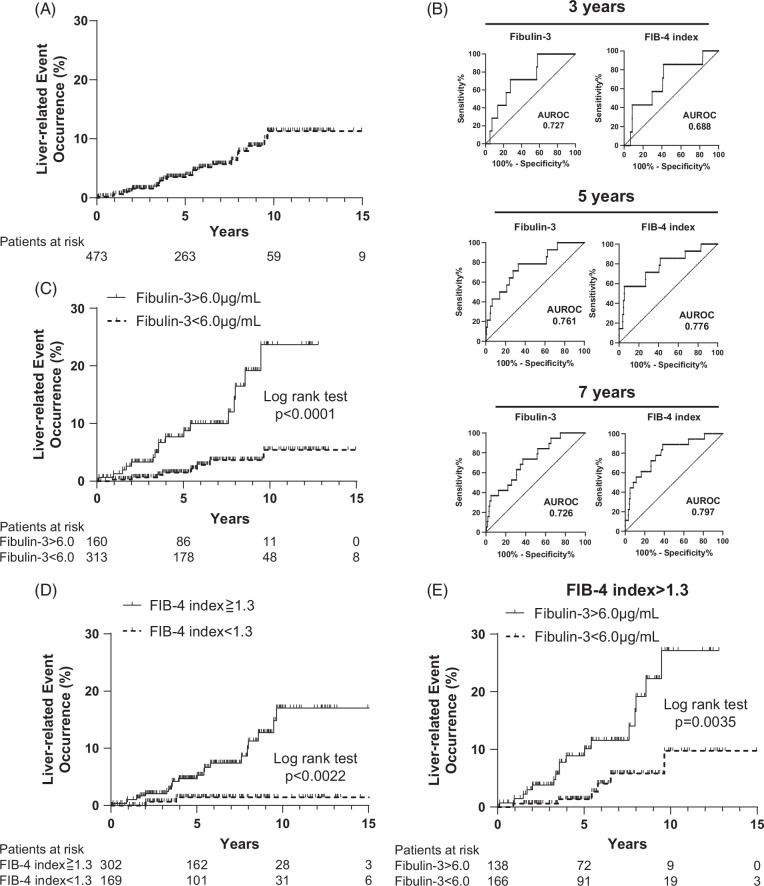
The serum Fibulin-3 concentration can predict liver-related events in patients with MASLD. (A) K-M curves for liver-related events in 473 patients with MASLD. (B) ROC curves of the ability of the serum Fibulin-3 concentration and FIB-4 index to predict liver cancer occurrence at 3, 5, and 7 years. (C) K-M curves for liver-related events in 473 patients with MASLD divided into 2 groups: serum Fibulin-3 >6.0 µg/mL and Fibulin-3 <6.0 µg/mL. (D) K-M curves for liver cancer occurrence and decompensation event occurrence in 471 patients with MASLD divided into 2 groups: FIB-4 index >1.3 and <1.3. (E) K-M curves for liver-related events occurring in 304 patients with MASLD with an FIB-4 index >1.3 according to the following 2 groups: serum Fibulin-3 >6.0 µg/mL and Fibulin-3 <6.0 µg/mL. Cutoff values were determined by the Youden index. The difference in cumulative event rates between the 2 groups was assessed by the log-rank test. Abbreviations: FIB-4, Fibrosis-4; K-M curve, Kaplan-Meier curve; MASLD, metabolic dysfunction–associated steatotic liver disease.

Next, we investigated whether the serum Fibulin-3 concentration can serve as a predictor of liver-related events, including liver cancer and decompensation, in patients with MASLD. The mean follow-up time after serum collection in cohort 2 was 6.2 years. During the observation period, 25 patients developed liver-related events. The incidence rates of liver-related events at 3, 5, and 7 years were 1.6%, 3.5%, and 5.7%, respectively, in this cohort (Figure 3A). We analyzed factors associated with liver-related events with a Cox proportional hazards model (Table 3). A lower platelet count, lower serum albumin concentration, higher alpha-fetoprotein, lower low-density lipoprotein cholesterol, higher type IV collagen 7S, higher hyaluronic acid, higher M2BPGi, higher FIB-4 index, and higher serum Fibulin-3 concentration were associated with an increased risk according to univariate analysis (Table 3). Considering that there were 25 liver-related events in this cohort, we selected the following 3 parameters for multivariate Cox regression models: hyaluronic acid concentration, FIB-4 index, and serum Fibulin-3 in model 1; type IV collagen 7S, FIB-4 index, and serum Fibulin-3 in model 2; and M2BPGi, FIB-4 index, and serum Fibulin-3 in model 3. A higher FIB-4 index and higher serum Fibulin-3 concentration were independently associated with an increased risk of liver-related events in each model (Table 3).

**TABLE 3:** HRs for liver-related events in 476 patients with biopsy-proven MASLD

	Univariate analysis		Multivariate analysis					
			Model 1		Model 2		Model 3	
	HR (95% CI)	*p*	HR (95% CI)	*p*	HR (95% CI)	*p*	HR (95% CI)	*p*
Age (y)	1.03 (1.00–1.07)	0.054						
Sex (male/female)	0.94 (0.42–2.09)	0.88						
BMI (kg/cm^2^)	1.02 (0.93–1.11)	0.68						
Diabetes mellitus (yes/no)	1.23 (0.56–2.71)	0.61						
Plt (×10^3^/µL)	0.85 (0.79–0.91)	7.5×10^-6^						
Alb (g/dL)	0.21 (0.09–0.49)	4.0×10^-4^						
T-Bil (mg/dL)	1.84 (0.73–4.64)	0.2						
AST (U/L)	1.01 (1.00–1.02)	0.130						
ALT (U/L)	1.00 (0.99–1.01)	0.65						
γGTP (U/L)	1.00 (1.00–1.01)	0.65						
AFP (ng/mL)	1.03 (1.00–1.06)	0.034						
HbA1c (%)	0.82 (0.56–1.21)	0.32						
TG (mg/dL)	1.00 (0.99–1.00)	0.20						
LDL-Chol (mg/dL)	0.98 (0.97–0.99)	0.003						
Type 4 collagen 7S (ng/mL)	1.34 (1.16–1.55)	7.7×10^-5^			1.12 (0.93–1.34)	0.23		
Hyaluronic acid (ng/mL)	1.002 (1.001–1.002)	1.2×10^-7^	1.00 (0.999–1.001)	0.706				
M2BPGi (COI)	1.77 (1.28–2.45)	5.2×10^-4^					1.26 (0.79–2.01)	0.33
FIB-4 index	1.56 (1.33–1.83)	4.6×10^-8^	1.23 (1.08–1.39)	0.0013	1.38 (1.13–1.68)	0.0018	1.39 (1.13–1.72)	0.002
Serum Fibulin-3 (μg/mL)	1.32 (1.19–1.45)	4.1×10^-8^	1.41 (1.16–1.71)	0.0062	1.20 (1.06–1.37)	0.0046	1.24 (1.09–1.40)	0.00077

*Note*: Independent factors associated with liver-related events were examined using Cox proportional hazards models. Considering that there were 25 liver-related events in this cohort, the following 3 parameters were selected for multivariate Cox regression models: hyaluronic acid concentration, FIB-4 index, and serum Fibulin-3 were used in model 1; type IV collagen 7S, FIB-4 index, and serum Fibulin-3 were used in model 2; and M2BPGi, FIB-4 index, and serum Fibulin-3 were used in model 3.

Abbreviations: γGTP, Gammna-glutamyl transpeptidase; AFP, alpha-fetoprotein; FIB-4, Fibrosis-4; LDL-Chol, low-density lipoprotein cholesterol; MASLD, metabolic dysfunction–associated steatotic liver disease; TG, triglyceride.

### The serum Fibulin-3 concentration and FIB-4 index can predict liver-related events in patients with MASLD

ROC curves predicting the occurrence of liver-related events within 3, 5, and 7 years were calculated for the serum Fibulin-3 concentration, and the AUROCs were 0.727, 0.761, and 0.726, respectively (Figure 3B). The AUROCs for the FIB-4 index were 0.688, 0.776, and 0.797, respectively; moreover, there were no significant differences between the ROC curves for the serum Fibulin-3 concentration and that for the FIB-4 index (by the DeLong test: Figure 3B). We set the cutoff value for the serum Fibulin-3 concentration at 6.0 µg/mL by referencing the Youden index,^33^ which was calculated using the AUROCs to predict liver-related events occurring within 3, 5, and 7 years: 6.4, 5.9, and 5.7, respectively. The occurrence rate of liver-related events at 5 years was 7.7% in patients with high serum Fibulin-3 levels (>6.0 µg/mL) and 1.5% in patients with low serum Fibulin-3 levels (<6.0 µg/mL) (Figure 3C), and there was a significant difference in the incidence of liver cancer between these 2 groups (log-rank test *p*<0.0001). With respect to the FIB-4 index, we set a cutoff value of 1.3, which is widely used as the primary screening for NAFLD diagnosis.^8^ Patients with a high FIB-4 index (>1.3) more frequently developed liver-related events than patients with a low FIB-4 index (<1.3), who rarely developed liver-related events (Figure 3D). Among patients with a high FIB-4 index (>1.3), those with high serum Fibulin-3 levels (>6.0 µg/mL) more frequently developed liver-related events than did patients with low serum Fibulin-3 levels (<6.0 µg/mL). The occurrence rates of liver-related events at 5 years were 8.9% and 1.4% in patients with high and low serum Fibulin-3 levels, respectively (Figure 3E). When liver-related events were analyzed separately for liver cancer and decompensation, patients with high serum Fibulin-3 or a high FIB-4 index developed each event at a significantly greater rate than did others (Supplemental Figure S3A, http://links.lww.com/HC9/A893, Supplemental Figure S3B, http://links.lww.com/HC9/A893). The FIB-4 index is known to have decreased diagnostic performance in the elderly.^34^ Therefore, we examined the ROC curve for the occurrence of liver-related events within 5 years in cohort 2 of patients aged 65 years or older. The AUROC of FIB-4 index was 0.673, which was lower than that of the overall cohort, while the AUROC of Fibulin-3 was 0.747, which was the same as that of the overall cohort (Supplemental Figure S3C, http://links.lww.com/HC9/A893). Serum Fibulin-3 may be effective in predicting the occurrence of liver-related events even in elderly patients with reduced diagnostic ability of FIB-4 index.

### A high serum Fibulin-3 concentration is validated to be a predictor of liver-related events in patients with MASLD

**FIGURE 4:**
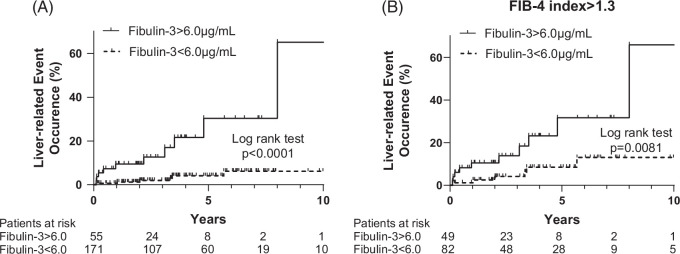
The serum Fibulin-3 concentration was validated to be a predictor of liver-related events in patients with MASLD. (A) K-M curves for liver-related events in 226 patients with MASLD divided into 2 groups: serum Fibulin-3 >6.0 µg/mL and Fibulin-3 <6.0 µg/mL. (B) K-M curves for liver cancer occurrence in 131 patients with MASLD syndrome with an FIB-4 index >1.3 according to the following 2 groups: serum Fibulin-3 >6.0 µg/mL and Fibulin-3 <6.0 µg/mL. The difference in cumulative event rates between the 2 groups was assessed by the log-rank test. Abbreviations: FIB-4, Fibrosis-4; K-M curve, Kaplan-Meier curve; MASLD, metabolic dysfunction–associated steatotic liver disease.

To validate the utility of Fibulin-3 as a liver-related biomarker for MASLD, we used a clinically diagnosed MASLD cohort that included patients who did not undergo liver biopsy (cohort 3). Cohort 3 included 226 patients with MASLD syndrome; the mean Fibulin-3 concentration was 4.8 µg/mL, and 16 patients developed liver-related events during the observation period (Supplemental Table S2, http://links.lww.com/HC9/A893). In cohort 3, the occurrence rates of liver-related events at 1, 3, and 5 years were 9.4%, 12.6%, and 30.3%, respectively, in patients with high serum Fibulin-3 levels (>6.0 µg/mL) and 0.6%, 2.0%, and 4.1%, respectively, in patients with low serum Fibulin-3 levels (<6.0 µg/mL) (Figure 4A). Among patients with a high FIB-4 index (>1.3), those with high serum Fibulin-3 levels (>6.0 µg/mL) more frequently developed liver-related events than did those with low serum Fibulin-3 levels (<6.0 µg/mL) (Figure 4B). When liver-related events were analyzed separately for liver cancer and decompensation, patients with high serum Fibulin-3 levels (>6.0 µg/mL) developed each event at a significantly greater rate than patients with low serum Fibulin-3 levels (<6.0 µg/mL) (Supplemental Figure S4A, http://links.lww.com/HC9/A893, Supplemental Figure S4B, http://links.lww.com/HC9/A893).

## DISCUSSION

In the present study, proteomic analysis of serum EVs revealed a novel biomarker, Fibulin-3, that increases with liver fibrosis and can predict liver-related events in patients with MASLD. The FIB-4 index has been used as a noninvasive fibrosis marker in primary screening for the diagnosis of NAFLD.^8^ Hyaluronic acid, type IV collagen 7S, and M2BPGi are reported to be fibrosis markers useful for differentiating NAFLD from advanced fibrosis.^35^,^36^ According to our multivariate analysis that included these factors, higher serum Fibulin-3 levels were identified as an independent risk factor for the occurrence of liver-related events in patients with MASLD, in addition to a higher FIB-4 index. The predictive value of Fibulin-3 for liver-related events was equivalent to that of the FIB-4 index, and that was not reduced even in the elderly group. Furthermore, since the serum Fibulin-3 concentration was able to stratify the occurrence of liver-related events even in the population with an FIB-4 index of 1.3 or higher, serum Fibulin-3 is useful for further screening of high-risk groups after primary screening with the FIB-4 index. As these indicate, Fibulin-3 is likely a valuable noninvasive marker for MASLD.

The number of serum EVs increases with the progression of NAFLD,^37^ and the protein profile of serum EVs from patients with NAFLD is also different from that from healthy individuals.^38^ We used proteomic analysis of serum EVs but not whole-serum samples to explore this topic. According to proteomic analyses of NAFLD patient sera, serum Fibulin-3 has not previously been identified as a candidate biomarker.^15^ It is assumed that usual serum proteome analysis may not be able to detect Fibulin-3 because the amount of Fibulin-3 secreted into serum is very low. Although protein levels in EVs do not necessarily correlate with those in serum, some proteins do, and the comprehensive search for serum EVs using proteomic analysis is a useful method for the discovery of new serum biomarkers.

The gene encoding Fibulin-3 is EFEMP1. Reports have shown that single-nucleotide polymorphisms in EFEMP1 are associated with the development of biliary atresia.^39^,^40^ Single-nucleotide polymorphisms in EFEMP1 are also associated with fat distribution in the body^41^ and with abdominal age.^42^ Although EFEMP1 is expressed in all organs of the body, serum Fibulin-3 in patients with MASLD is higher in cases with advanced liver fibrosis (Figure 2A) and high intrahepatic inflammation (Figure 2C), and there was a correlation between serum Fibulin-3 and intrahepatic Fibulin-3 expression (Supplemental Figure S2D, http://links.lww.com/HC9/A893) in our study. These results indicate that serum Fibulin-3 generally reflects secretion from the liver. Other reports have also shown that EFEMP1 is one of the genes that is markedly upregulated in the liver of patients with NAFLD with advanced fibrosis.^43^,^44^ And EFEMP1-expressing cells in NAFLD liver have been reported to be hepatocytes, vascular endothelial cells, and fibroblasts.^44^ Physiologically, Fibulin-3 plays an important role in the formation of elastic fibers.^45^ Fibulin-3 has also been linked to various organ fibrosis. It contributes to the promotion of myocardial fibrosis in the heart.^46^ An association between EFEMP1 single-nucleotide polymorphisms and constrictive lung disease including pulmonary fibrosis has also been reported.^47^ Fibulin-3 interacts with matrix metalloproteinases^45^ that contribute to collagen degradation and has a regulatory effect on TGF-β,^48^ which is involved in the activation of HSCs in the liver. In view of these points, it is possible that Fibulin-3 secreted by EFEMP1-expressing cells may play some role in liver fibrosis in MASLD.

Regarding the relationship between Fibulin-3 and liver cancer, there are reports that Fibulin-3 inhibits the development of HCC^49^ and that patients with HCC with low Fibulin-3 expression have a poor prognosis.^50^ Although these reports seemingly contradict our results that serum Fibulin-3 levels are associated with a significantly greater incidence of liver cancer (Supplemental Figure S3A, http://links.lww.com/HC9/A893, Supplemental Figure S4A, http://links.lww.com/HC9/A893), we focused on Fibulin-3 before the development of liver cancer and others focused on Fibulin-3 in liver cancer. Whether Fibulin-3 has any significance in the pathogenesis of MASLD is currently unknown, and further investigations are needed.

In conclusion, by performing proteomic analysis of serum EVs, we found that the biomarker Fibulin-3 is a good predictor of the occurrence of liver-related events in patients with MASLD. Patients with MASLD who should be followed up regularly can be identified by measuring serum Fibulin-3 without performing liver biopsy.

## Supplementary Material

SUPPLEMENTARY MATERIAL
