# Supplementary material for: Proteomic analysis of serum extracellular vesicles reveals Fibulin-3 as a new marker predicting liver-related events in MASLD
Source: Hepatol Commun. 2024 Jun 3;8(6):e0448. doi: 10.1097/HC9.0000000000000448 (PMC11150025; doi:10.1097/HC9.0000000000000448)
Supplement: SUPPLEMENTARY MATERIAL [file hc9-8-e0448-s001.pdf]

Supplemental Figure 1

(A) Cohort1

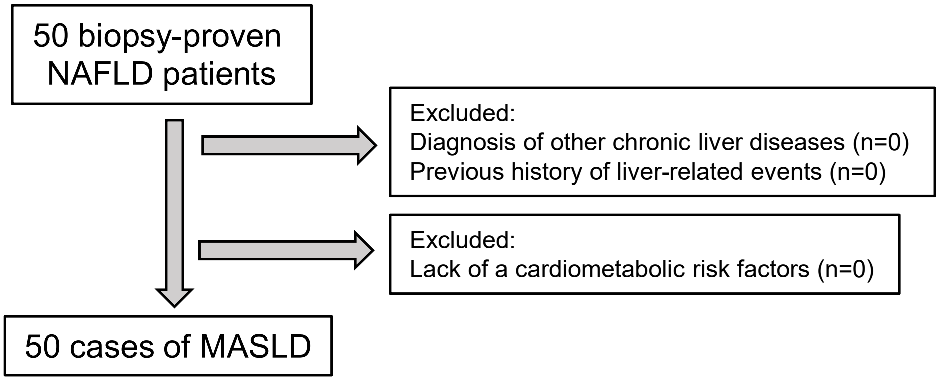

(B) Cohort 2

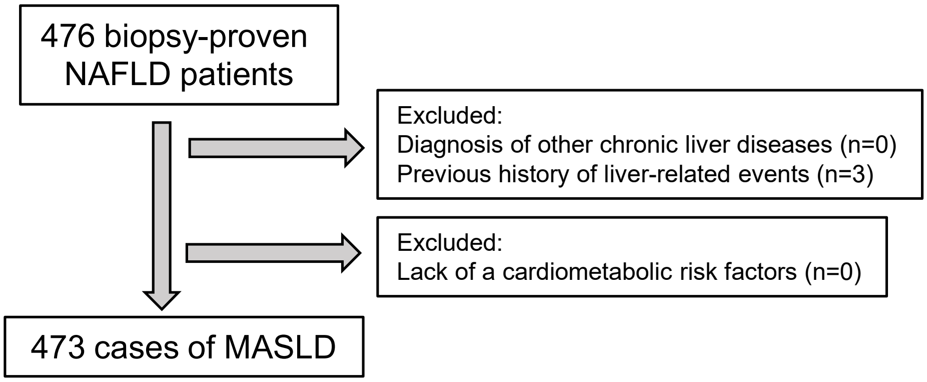

(C) Cohort 3

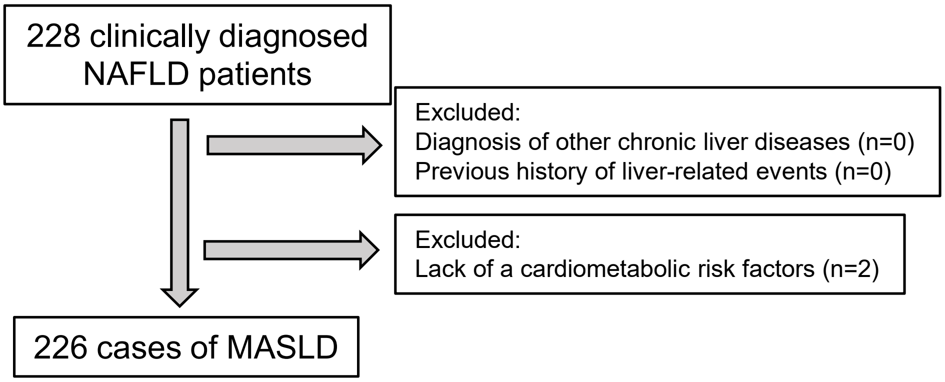

Supplemental Figure 2

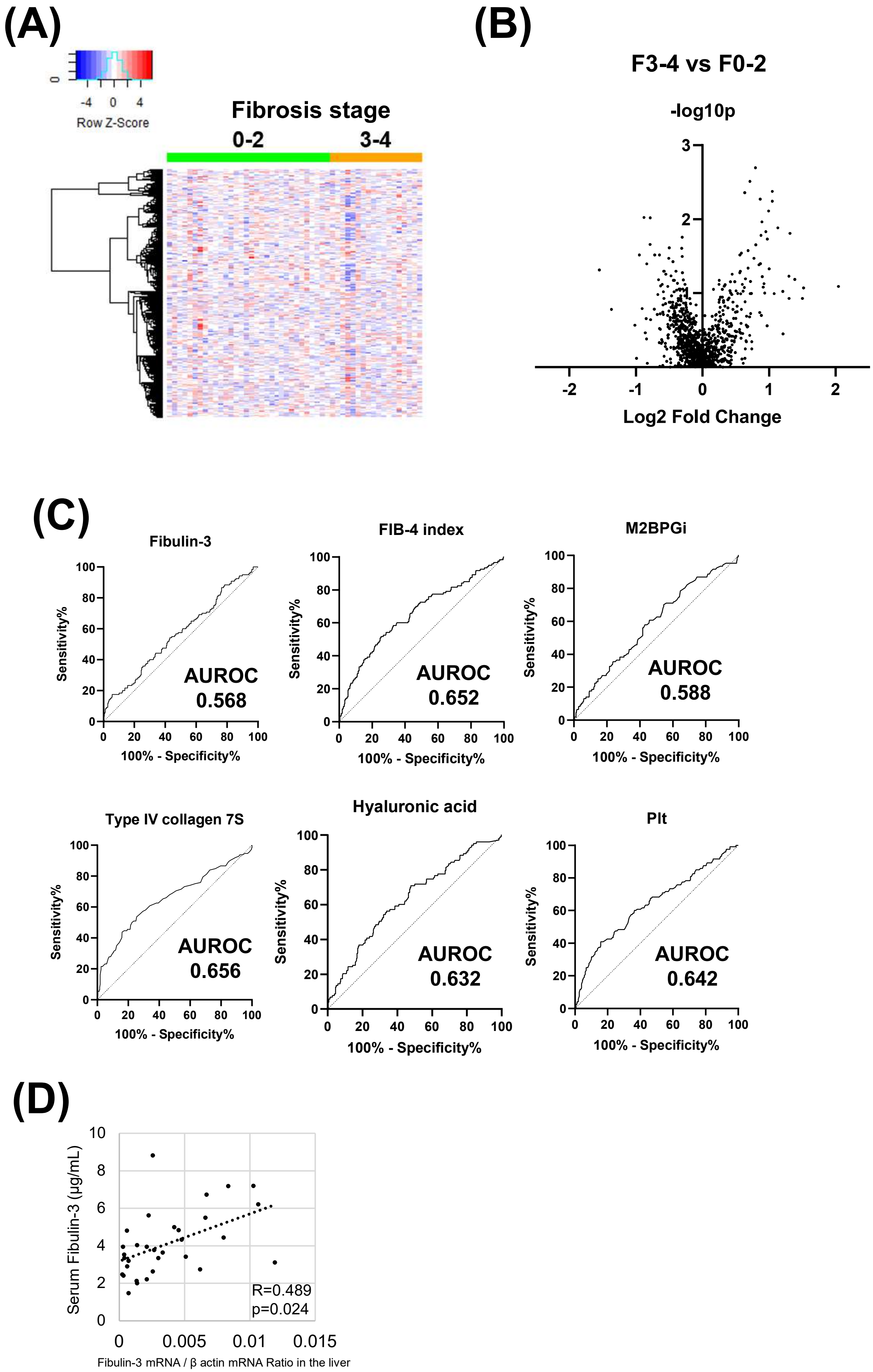

Supplemental Figure 3

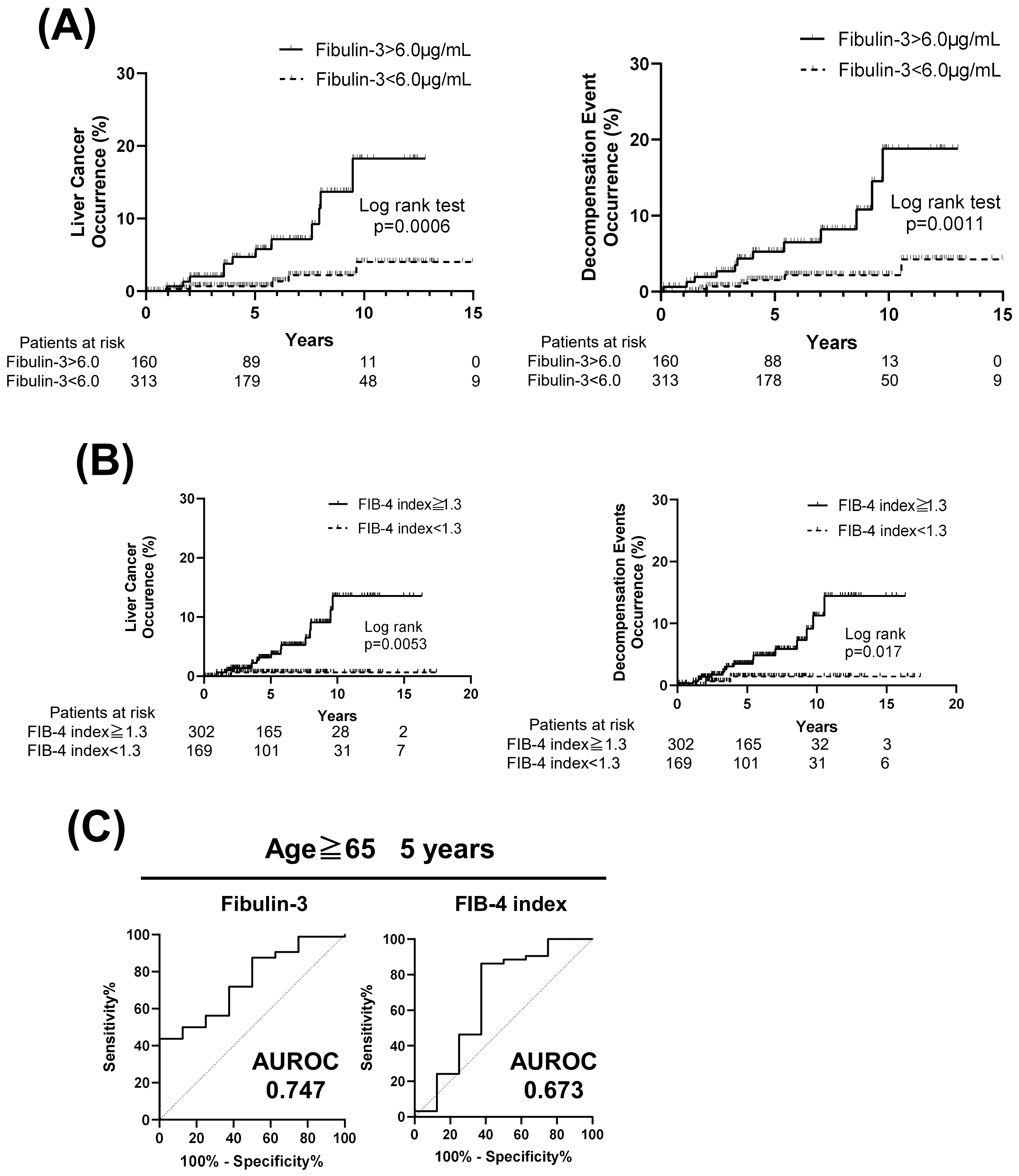

Supplemental Figure 4

(A)

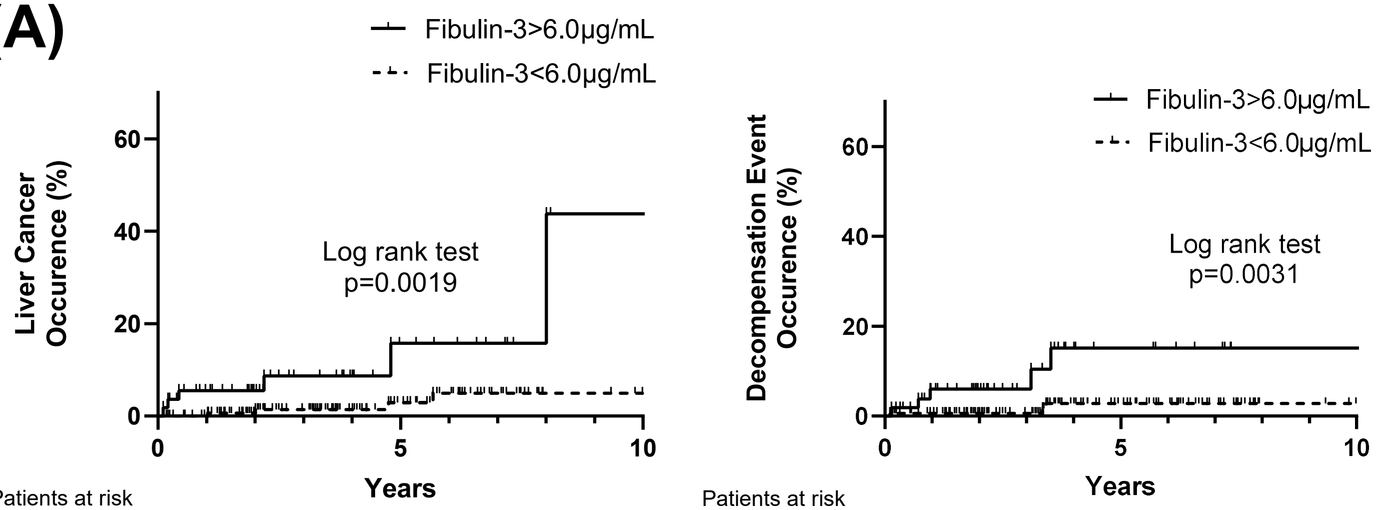

Patients at risk

Fibulin-3>6.0	55	26	11	3	1
Fibulin-3<6.0	171	108	60	19	10

Patients at risk

Fibulin-3>6.0	55	25	9	1	1
Fibulin-3<6.0	171	108	60	20	10

Supplemental Table 1

	All (N=50)	F0-F2 (N=32)	F3-F4 (N=18)	p value
Age (years)	60.1 [28-85]	56.8 [28-80]	66 [50-85]	<0.001
Sex (Male/Female)	20/30	13/19	7/11	0.9
Height (cm)	159.4 [145-178]	160.2 [145-176]	158.1 [145-178]	0.4
Weight (kg)	70 [46.9-121]	69.7 [48.6-100]	70.5 [46.9-121]	0.88
BMI (kg/cm^2)	27.5 [20.7-51.7]	27.1 [20.7-36.6]	28.1 [20.8-51.7]	0.59
Diabetes mellitus (Yes/No)	21/29	10/22	11/7	0.04
Hypertension (Yes/No)	24/26	12/20	12/6	0.048
Dyslipidemia (Yes/No)	22/28	14/18	8/10	0.96
Plt (x10^3/μl)	215.6 [57-514]	242.6 [63-514]	167.6 [57-262]	<0.001
Alb (g/dl)	4.2 [2.8-4.8]	4.3 [3.2-4.8]	4.1 [2.8-4.6]	0.18
T-Bil (mg/dl)	0.7 [0.2-1.4]	0.7 [0.2-1.4]	0.7 [0.3-1.4]	0.75
AST (U/l)	64.3 [20-214]	65.3 [22-214]	62.6 [20-165]	0.8
ALT (U/l)	81.3 [15-251]	90.1 [18-181]	65.7 [27-251]	0.12
γGTP (U/l)	108.7 [20-521]	121 [20-521]	86.8 [20-189]	0.15
ChE (mg/dl)	342.2 [199-495]	370.1 [257-495]	288.2 [199-387]	<0.001
AFP (ng/ml)	5.1 [2-13]	4.7 [2-13]	6.0 [3-11]	0.055
Homa-IR	5.0 [0.1-59]	3.8 [0.6-14.5]	7.2 [0.1-59]	0.3
HbA1c (%)	6.3 [4.9-10.4]	6.4 [5.1-10.4]	6.3 [4.9-8.2]	0.83
T-Chol (mg/dl)	193.5 [119-272]	200.5 [119-272]	181.1 [139-225]	<0.001
TG (mg/dl)	157.3 [30-358]	169 [65-358]	136.6 [30-266]	0.13
HDL-Chol (mg/dl)	47.5 [24-95]	49.2 [29-95]	44.6 [24-63]	0.23
LDL-Chol (mg/dl)	114.6 [71.4-184.2]	117.5 [77-184.2]	109.2 [71.4-144.4]	0.29
Type 4 Collagen 7S (ng/ml)	6.8 [3.6-13]	6.4 [3.6-13]	7.4 [4.8-12]	0.14
Hyaluronic acid (ng/ml)	102.3 [9-384]	70.2 [9-275]	166.5 [39-384]	0.008
M2BPGi (COI)	1.3 [0.38-4.82]	1.1 [0.38-2.68]	1.9 [0.67-4.82]	0.041
FIB-4 index	2.6 [0.5-9.9]	1.9 [0.5-5.5]	3.9 [1.1-9.9]	<0.001
Steatosis (0/1/2/3)	0/16/14/20	0/7/10/15	0/9/4/5	0.12
Inflammation (0/1/2/3)	19/20/11/0	12/12/8/0	7/8/3/0	0.78
Ballooning (0/1/2)	37/12/1	25/6/1	12/6/0	0.41
NAS score (0/1/2/3/4/5/6/7/8)	0/7/11/10/10/11/1/0/0	0/5/6/4/7/9/1/0/0	0/2/5/6/3/2/0/0	0.38
Fibrosis stage (0/1/2/3/4)	6/17/9/12/6	6/17/9/0/0	0/0/0/12/6	<0.001

Supplemental Table 2

	All (N=226)	Fibulin-3<6.0μg/mL (N=171)	Fibulin-3>6.0μg/mL (N=55)	p value
Age (years)	53 [17-89]	50 [17-89]	64 [37-83]	<0.0001
Sex (Male/Female)	107/119	88/83	19/36	0.028
BMI (kg/cm^2)	29.1 [17.6-64.4]	29.2 [17.6-64.4]	29.1 [18.8-46.0]	0.89
Diabetes mellitus (Yes/No)	89/137	64/107	25/30	0.28
Plt (x10^3/μl)	212 [33-666]	224 [33-396]	173 [49-666]	0.00032
Alb (g/dl)	4.3 [2.7-5.5]	4.4 [3.3-5.5]	4.0 [2.7-4.9]	<0.0001
T-Bil (mg/dl)	0.9 [0.2-3.5]	0.8 [0.2-3.5]	1.0 [0.3-2.7]	0.0020
AST (U/l)	53 [14-241]	53 [14-241]	55 [19-186]	0.67
ALT (U/l)	73 [10-713]	79 [10-713]	55 [12-296]	0.011
γGTP (U/l)	86 [14-676]	88 [14-676]	79 [17-530]	0.47
AFP (ng/ml)	6.0 [1.3-198]	4.1 [1.3-27]	11.5 [1.8-198]	0.10
HbA1c (%)	6.5 [0.3-13.6]	6.5 [0.3-13.6]	6.3 [4.4-9.0]	0.18
TG (mg/dl)	186 [39-892]	197 [39-642]	153 [41-892]	0.047
LDL-Chol (mg/dl)	118 [29-227]	124 [47-227]	99 [29-147]	0.00018
Type 4 Collagen 7S (ng/ml)	5.6 [2.1-15.6]	5.1 [2.1-15.6]	7.3 [3.3-14.8]	<0.0001
Hyaluronic acid (ng/ml)	92 [10-741]	64 [10-614]	176 [10-741]	0.00022
M2BPGi (COI)	1.3 [0.3-7.6]	1.0 [0.3-6.7]	2.2 [0.4-7.6]	0.00072
FIB-4 index	2.12 [0.12-12.01]	1.65 [0.12-8.73]	3.60 [0.58-12.01]	<0.0001
Liver cancer occurrence (Yes/No)	10/216	4/167	6/49	0.0071
Decompensation event (Yes/No)	8/218	3/168	5/50	0.010
Liver-related event (Yes/No)	16/210	6/166	10/45	0.00021
Observation period (years)	4.0 [0.2-13.4]	4.2 [0.2-13.4]	3.3 [0.2-10.4]	0.018
Fibulin-3 (μg/ml)	4.8 [1.5-18.6]	3.5 [1.5-6.0]	8.9 [6.0-18.6]	<0.0001
